# From Endometriosis to Endometriosis-Associated Ovarian Cancer: Molecular Mechanisms, Risk Stratification and Clinical Implications

**DOI:** 10.3390/cancers18081233

**Published:** 2026-04-14

**Authors:** Felice Sorrentino, Luigi Nappi, Laura Vona, Lorenzo Vasciaveo, Maria Rosaria Campitiello, Paola Vitrani, Gloria Taurino, Raffaele Tinelli, Elvira Grandone

**Affiliations:** 1 Department of Medical and Surgical Sciences, Institute of Obstetrics and Gynaecology, University of Foggia, 71121 Foggia, Italy; felice.sorrentino@unifg.it (F.S.); luigi.nappi@unifg.it (L.N.); laura.vona@unifg.it (L.V.); lvasciaveo@ospedaliriunitifoggia.it (L.V.); mr.campitiello@sanita.it (M.R.C.); paola_vitrani.578398@unifg.it (P.V.); 2 Fondazione I.R.C.C.S. “Casa Sollievo della Sofferenza”, 71013 San Giovanni Rotondo, Italy; g.taurino@operapadrepio.it; 3 Department of Obstetrics and Gynecology, “Valle d’Itria” Hospital, 74015 Martina Franca, Italy; raffaele.tinelli@asl.taranto.it

**Keywords:** endometriosis, endometriosis-associated ovarian cancer, molecular pattern, inflammation

## Abstract

Endometriosis is a chronic estrogen-dependent disorder affecting approximately 10% of women of reproductive age. Increasing epidemiological and molecular evidence indicates that it may represent a precursor condition for a subset of ovarian malignancies collectively defined as endometriosis-associated ovarian cancer (EAOC), predominantly endometrioid and clear cell carcinomas. Malignant transformation is driven by the interplay between chronic inflammation, oxidative stress, and local hyperestrogenism within the endometriotic microenvironment. Recurrent hemorrhage and persistent immune activation further promote genomic instability and clonal expansion. Shared somatic mutations have been identified in both atypical endometriosis and adjacent carcinomas, supporting a model of stepwise tumorigenesis. Dysregulation of signaling pathways and epigenetic mechanisms, including microRNA alterations, further contribute to tumor development. Although the absolute risk of malignant transformation remains low, women with ovarian endometriosis and deep infiltrating disease show an increased risk of ovarian cancer. EAOC is frequently diagnosed at earlier stages and generally demonstrates a more favorable prognosis than high-grade serous carcinoma, although clear cell histotypes may exhibit chemoresistance and distinct molecular vulnerabilities. This review summarizes current evidence on the pathogenesis, molecular mechanisms, and clinical implications of EAOC, highlighting future strategies for risk stratification and personalized surveillance.

## Simple Summary

Endometriosis is increasingly recognized as more than a benign gynecological disorder, as evidence suggests it can act as a precursor to specific ovarian cancers, particularly endometrioid ovarian carcinoma and clear cell carcinoma. Chronic inflammation, prolonged estrogen exposure, and immune dysregulation create a microenvironment that promotes genetic alterations and malignant transformation. Molecular studies have identified shared driver mutations in both endometriotic lesions and associated tumors, supporting a model of clonal progression from benign tissue to cancer. Although the overall risk of ovarian cancer remains relatively low, women with endometriosis have a higher relative risk for these histological subtypes. Future research should focus on early detection through non-invasive biomarkers, improved risk stratification, and the development of targeted therapies to enable more personalized surveillance and treatment strategies.

## 1. Introduction

Endometriosis is a chronic, estrogen-dependent gynecological disorder characterized by the presence of ectopic endometrial-like tissue—comprising both glandular epithelium and stroma—outside the uterine cavity [1]. While the ovaries, pelvic peritoneum, and uterosacral ligaments are the most frequently affected sites, lesions can manifest in extra pelvic locations, including the gastrointestinal tract, bladder, diaphragm, and, in rare instances, the thoracic cavity or central nervous system [2]. These ectopic implants remain hormonally active, undergoing cyclic proliferation and hemorrhage in response to ovarian steroids. This persistent activity triggers a cascade of chronic inflammation, reactive oxygen species (ROS) production, and subsequent fibrosis. These mechanisms underpin the classic clinical presentation of chronic pelvic pain, dysmenorrhea, and infertility [3].

Endometriosis is estimated to affect approximately 10% of women of reproductive age, with its prevalence escalating to 30–50% among those presenting with infertility or chronic pelvic pain [4]. Clinical presentation typically peaks between 25 and 35 years. However, the disease is increasingly recognized in adolescents—particularly those with obstructive congenital uterine anomalies [5]—and may persist or emerge in the postmenopausal period, often driven by exogenous hormone replacement or peripheral aromatization of androgens [6].

Despite its significant prevalence, the diagnostic trajectory is often marred by a latency period of 7–10 years from the onset of symptoms. This delay is attributed to the marked phenotypic heterogeneity of the disease and the frequent societal normalization of menstrual pain in young women [7]. Symptomatology is diverse; while pain is classically cyclic and synchronized with hormonal fluctuations, it often transitions into a non-cyclic, persistent state in advanced stages as a result of central sensitization and nerve infiltration [8]. Furthermore, cyclic hemorrhage at extra pelvic sites serves as a pathognomonic sign of atypical endometriosis [9]. Depending on the anatomical involvement, patients may present with catamenial epilepsy, hemoptysis, hematochezia, or catamenial pneumothorax [10,11]. Beyond physical morbidity, the condition exerts a profound psychosocial burden, frequently leading to diminished professional productivity, impaired social engagement, and a significant reduction in overall quality of life [12,13].

## 2. Materials and Methods

A comprehensive literature search was conducted using PubMed, Scopus, and Web of Science, employing the keywords: *endometriosis; endometriosis-associated ovarian cancer; molecular pattern; inflammation*. English-language articles, including original research, reviews, and case reports, were considered. Studies were selected based on their relevance to molecular mechanisms, clinical risk factors, diagnostic evaluation, and management strategies of endometriosis-associated ovarian cancer. Titles, abstracts, and full texts were screened to ensure a focused synthesis linking molecular insights with practical clinical applications.

## 3. Etiopathogenetic Theories: From Classical Hypotheses to the Endometriotic Disease Theory (EDT)

While several historical models attempt to explain the origin of endometriosis, no single hypothesis comprehensively accounts for its full clinical spectrum.

The main pathogenic theories can be broadly categorized into three mechanisms. The Retrograde Menstruation theory [14] remains the cornerstone for understanding peritoneal disease, suggesting the reflux of viable endometrial fragments through the fallopian tubes. In parallel, this is complemented by the Coelomic Metaplasia theory [15], which proposes the transformation of peritoneal mesothelium into endometrial-like tissue, and the Lymphatic/Hematogenous Dissemination theory [16], which accounts for rare extra pelvic implantations through a mechanism reminiscent of metastatic spread.

However, these models alone do not fully explain disease persistence and progression. Modern research suggests that they represent initiating events acting on a predisposed molecular background. This concept is summarized in the Endometriotic Disease Theory (EDT), which reframes endometriosis as a systemic disorder driven by genetic and epigenetic alterations [17].

Within this framework, epigenetic dysregulation plays a central role. Mechanisms including DNA methylation, histone modifications, and microRNA (miRNA) imbalance alter gene expression without modifying the underlying DNA sequence, generating a stable “cellular memory” that supports ectopic cell survival [14]. A primary driver of this pathological phenotype is aberrant promoter methylation. For instance, hypermethylation of the HOXA10 gene—a key regulator of endometrial differentiation—leads to its transcriptional silencing, impairing normal decidualization and uterine receptivity, and thus directly contributing to the high rates of infertility associated with the disease [17]. Conversely, hypomethylation of the CYP19A1 gene (encoding aromatase) results in its overexpression, transforming ectopic lesions into autonomous estrogen-producing sites and sustaining local proliferation [17,18].

Post-transcriptional regulation further amplifies this effect. By binding to the 3’ untranslated regions of target messenger RNAs (mRNAs), miRNA can either promote mRNA degradation or inhibit translation by blocking ribosome access [14]. In the endometriotic microenvironment, the dysregulation of specific miRNA families often leads to the “de-repression” of oncogenic pathways. This allows for the overproduction of proteins involved in the epithelial–mesenchymal transition (EMT) and angiogenesis, such as VEGF, which grant the ectopic tissue its characteristic invasive capacity [14,19].

This epigenetic vulnerability is reinforced by a significant genetic component, evidenced by a seven-fold increase in risk among first-degree relatives [20]. This genetic–epigenetic synergy appears to govern the behaviour of endometrial and mesenchymal stem cells; their high plasticity and inherent resistance to apoptosis facilitate both their migration to and survival at ectopic sites [19].

Once implanted, these lesions establish a self-sustaining pro-inflammatory microenvironment characterized by activated macrophage infiltration and the continuous secretion of cytokines, such as TNF-α (Tumour necrosis factor-α) [21]. A shift in estrogen receptor, with ERβ (Estrogen Receptor β) predominance over ERα (Estrogen Receptor α) further promotes cell survival and resistance to apopstosis [18].

The clinical significance of this chronic state extends beyond pain and infertility. The repetitive cycle of intra-cystic hemorrhage leads to the accumulation of free iron, which, through the Fenton reaction, generates a massive influx of Reactive Oxygen Species (ROS). This persistent oxidative stress induces cumulative DNA damage and genomic instability, serving as the critical bridge that transforms a benign “epigenetic phenotype” into a malignant “mutational phenotype,” eventually manifesting as endometriosis-associated ovarian carcinoma (EAOC) [2].

## 4. Diagnosis of Endometriosis

The diagnostic trajectory for endometriosis remains challenging due to significant clinical heterogeneity and the absence of pathognomonic non-invasive markers, leading to a documented latency of up to a decade [1]. Current evidence-based strategies, as delineated in the ESHRE 2022 guidelines, advocate for a shift toward a multimodal diagnostic framework that prioritizes high-resolution imaging and clinical correlation over historical “surgical-only” paradigms (Table 1).

Transvaginal Ultrasound (TVUS) represents the primary diagnostic modality, exhibiting high sensitivity for the detection of ovarian endometriomas and deep infiltrating endometriosis (DIE) [22]. In cases of complex pelvic involvement or suspected extra-peritoneal extension, Magnetic Resonance Imaging (MRI) is indicated for precise anatomical mapping. Critically, the diagnostic yield of MRI is operator-dependent; thus, images should be evaluated by radiologists specialized in gynecological oncology and pelvic imaging within tertiary referral centers [23].

Despite advances in imaging, laparoscopy remains the definitive reference standard for comprehensive staging and histological verification. In the management of ovarian endometrioma, laparoscopic cystectomy—the excision of the cyst wall—is superior to simple drainage or ablation, as it provides the necessary tissue volume for meticulous histological screening to exclude atypical endometriosis or early-stage neoplastic transformation [24]. Regarding biochemical markers, the clinical utility of CA-125 remains limited by poor specificity; elevated levels are common in various benign inflammatory states, thus precluding its use as a primary diagnostic or screening tool [1,25].

**Table 1:** Diagnostic and Management Standards (adapted from ESHRE 2022 Guidelines).

Diagnostic Approach	Recommendation	Clinical Justification
First-line Imaging	Transvaginal ultrasound	Preferred initial tool due to high sensitivity and wide availability. Effectively detects endometriomas and deep infiltrating endometriosis of the posterior
Advanced Imaging	Expert-led MRI ^1^	Recommended when TVUS findings are inconclusive, or surgery is planned. Provides superior soft-tissue contrast for mapping extent of DIE, bowel involvement, and multifocal disease—critical for surgical planning [23].
Surgical Standard	Laparoscopic Cystectomy	Gold standard for ovarian endometriomas. Allows histological confirmation to exclude malignancy, offers lower recurrence rates compared to drainage/ablation, and may improve fertility outcomes [24].
Serum Markers	CA-125 (Limited use)	Not recommended as a standalone diagnostic tool. Elevated in many benign and malignant conditions, resulting in poor specificity. May have a supplementary role in monitoring disease activity or treatment response [25].

^1^ MRI: Magnetic Resonance Imaging.

## 5. Evolution of Classification Systems and Morphological Phenotypes

The inherent complexity and clinical diversity of endometriosis have necessitated the development of various staging systems, though no single tool comprehensively captures the disease’s full spectrum [26]. The revised American Society for Reproductive Medicine (rASRM) classification has served as the reference standard, categorizing disease into four stages (I–IV) based on anatomical distribution and adhesion density [27]. However, the rASRM system is increasingly scrutinized for its poor correlation with pain severity and its limited capacity to predict spontaneous pregnancy outcomes [28]. The transition from purely anatomical staging to functional and risk-based assessments is summarized in Table 2, which illustrates the diverse clinical priorities of contemporary classification tools.

The #Enzian classification provides superior mapping for Deep Infiltrating Endometriosis (DIE) by grading compartment-specific involvement [29], while the Endometriosis Fertility Index (EFI) integrates surgical data with functional reproductive assessments to provide more accurate fertility prognoses [27]. More recently, the AAGL 2021 Classification has introduced a staging approach centered on surgical complexity and operative risk [30].

Despite these advancements, anatomical maps alone do not fully reflect the biological “aggressiveness” of different lesions. Consequently, a morphological classification based on tissue infiltration depth is clinically essential [31], categorizing endometriosis into three distinct entities:

***Superficial Peritoneal Endometriosis (SPE):*** Implants penetrate less than 5 mm into the peritoneum. Despite their limited depth, these lesions remain highly active, inducing localized vascular proliferation and recurrent cyclic bleeding. Their varied clinical appearance—ranging from red and black to white plaques—serves as a chronological marker of lesion evolution, reflecting progressive hemosiderin accumulation and fibrotic matrix development.

***Ovarian Endometriosis (Endometrioma):*** Characterized by the formation of “chocolate cysts” containing hematic debris [32]. Beyond compromising ovarian reserve and fertility through local inflammatory pressure, endometriomas constitute the primary site of neoplastic transformation. The unique intra-cystic environment functions as a mutagenic incubator, establishing the endometrioma as the principal precursor lesion for EAOCs [33].

***Deep Infiltrating Endometriosis (DIE):*** Infiltrative growth exceeds 5 mm beneath the peritoneal surface [34], typically involving the rectovaginal septum, bowel, or bladder. Intense myofibroblastic proliferation and nerve entrapment explain the severe pain and social impairment associated with DIE [35].

**Table 2:** Comparative Overview of Endometriosis Classification Systems.

Classification System	Primary Focus	Key Features and Clinical Utility
rASRM ^1^	Anatomical Mapping	Stages I–IV based on lesion size/depth and adhesions. Standardized for documentation; poor correlation with pain and fertility outcomes [26,28]
Enzian	Deep Endometriosis	Compartment-specific grading. Useful for preoperative planning of complex infiltrative disease [29].
EFI ^2^	Reproductive Prognosis	Combines surgical findings with functional adnexal assessment. Predicts spontaneous conception rates after surgery [27].
AAGL 2021 ^3^	Surgical Complexity	Ranks disease based on operative difficulty. Correlates with operative time, risk, and clinical symptoms [30].
Morphological	Biological Phenotype	Distinguishes lesions by infiltration depth (<5 mm vs. >5 mm). Essential for oncogenic risk assessment and identifying EAOC precursors [31,33].

^1^ rASRM: revised American Society for Reproductive Medicine classification; ^2^ EFI: Endometriosis Fertility Index; ^3^ AAGL: American Association of Gynecologic Laparoscopists.

## 6. Endometriosis and Neoplastic Transformation: From Inflammation to Malignancy

Extensive epidemiological and molecular evidence has characterized endometriosis not merely as a benign gynecological disorder, but as a condition associated with an increased risk of specific malignancies, collectively termed endometriosis-associated ovarian cancer (EAOC). Although histologically benign, endometriosis is characterized by a chronic inflammatory milieu. This environment is driven by persistent hormonal stimulation, immune dysregulation, and the accumulation of reactive oxygen species (ROS), largely resulting from repeated hemorrhage and iron overload. Together, these factors contribute to the establishment of a permissive microenvironment that may favor neoplastic transformation.

Over time, these conditions promote cellular stress, DNA damage, and aberrant repair mechanisms, facilitating the transition from benign endometriotic lesions to atypical endometriosis—regarded as an intermediate precancerous state. This transformation is accompanied by the acquisition of somatic mutations and molecular alterations, ultimately leading to malignant phenotypes, most commonly clear cell carcinoma (CCC) and endometrioid ovarian carcinoma (EnOC).

**Figure 1:**
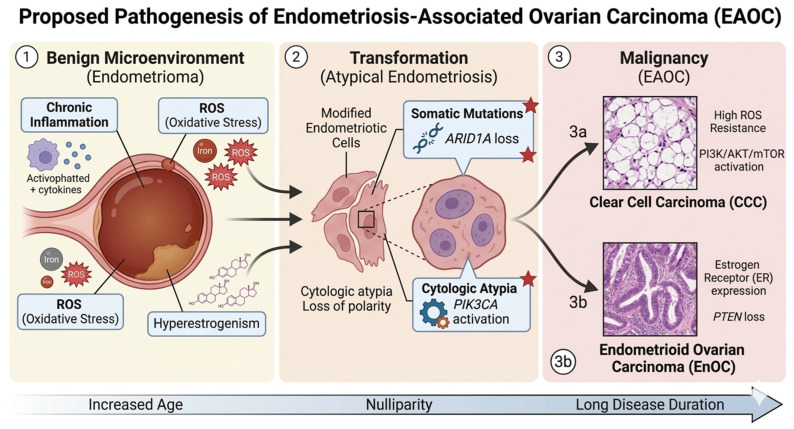
Proposed pathogenesis of endometriosis-associated ovarian carcinoma (EAOC). The figure was generated using Google Gemini (AI-assisted illustration) https://gemini.google.com/app?hl=it (accessed on 14 March 2026).

Figure 1 schematically summarizes this stepwise progression, highlighting the key biological processes and molecular events underlying the evolution from a benign endometriotic microenvironment to overt malignancy [36,37].

Beyond the ovary, Mendelian randomization [36,37] and meta-analyses [38] suggest a systemic vulnerability, with women exhibiting a moderately increased risk for other estrogen-dependent malignancies, including endometrial and breast cancers. However, the relationship is most definitive in ovarian carcinoma, where a precursor-product continuum is supported by the identification of transitional lesions (TLs) and atypical endometriosis [32,39]. This progression defines the “Endometriosis-Correlated Ovarian Cancer” (ECOC) paradigm, marking the juncture where benign epithelium acquires the genomic instability necessary for malignant escape [40].

### 6.1. Malignant Transformation: Risk Profiles and Clinical Indicators

Women with endometriosis face a nearly twofold increase in the relative risk of ovarian carcinoma [36]. This risk is markedly higher for specific histotypes, with a 3- to 4-fold increase for Endometrioid Ovarian Carcinoma (EnOC) and Clear Cell Carcinoma (CCC) [41,42]. To date, no reliable predictive molecular markers or clinical indicators have been identified to stratify which women with endometriosis are at greatest risk of malignant transformation. The identification of such markers would carry substantial clinical implications, potentially enabling risk-adapted active surveillance or timely prophylactic surgical intervention, with the ultimate goal of reducing disease-specific mortality.

Clinical “red flags” for malignant transformation include advanced age (>45–50 years), postmenopausal status, and nulliparity [43] (Table 3). Specifically, nulliparity may increase risk by up to fourfold, as it lacks the “protective” physiological hypoestrogenism and ovulatory arrest provided by pregnancy [38,41]. Conversely, the use of combined oral contraceptives significantly mitigates this risk—reducing it by approximately 30–50%—by limiting repetitive ovulatory injury and reducing cyclic estrogen exposure [4,36]. In the postmenopausal period, the risk peaks; while endometriosis typically regresses after menopause, the persistence of lesions suggests a subgroup with high cumulative estrogen exposure or inherent genomic vulnerability [39,44]. Notably, a whole-exome sequencing study of paired endometriosis and ovarian carcinoma samples reported a median interval of 10 years between endometriosis surgery and subsequent cancer diagnosis, with a positive correlation between the degree of clonal dominance—as measured by variant allele frequency—and the time to malignant transformation, suggesting that clonal evolution within endometriotic lesions may be a temporally extended process [45].

### 6.2. The Role of Somatic Driver Mutations

The molecular transition to EAOC is a multistep process mediated by the acquisition of somatic driver mutations, predominantly affecting the epithelial compartment [46]. Approximately one-third of benign endometriotic lesions have been reported to harbor somatic mutations in genes such as ARID1A, PIK3CA, PTEN, and KRAS, although prevalence rates vary across different studies and patient cohorts [46,47,48]. While these mutations confer survival advantages—such as resistance to apoptosis and enhanced angiogenesis—their presence in eutopic endometrium suggests that mutations alone are insufficient for malignancy without the “second hit” provided by the inflammatory microenvironment [48,49,50]. Variability in mutation frequencies underscores the heterogeneity of patient populations and study methodologies, and understanding which mutations confer the highest oncogenic risk may inform early detection strategies and guide individualized clinical monitoring.

Of particular clinical significance is the observation that iatrogenic endometriosis—such as lesions arising in surgical scars—also harbors somatic cancer-driver mutations, suggesting that the potential for malignant transformation is an intrinsic feature of endometrial tissue that may be transferred or induced during surgical procedures [45]. This finding underscores the importance of meticulous surgical technique and thorough follow-up in patients with atypical endometriotic lesions.

### 6.3. Molecular Patterns of Clonal Divergence in Endometriosis-Associated Cancers

The shared mutational landscape between precursor lesions and adjacent carcinomas supports a model of clonal progression, wherein endometriotic cells undergo stepwise evolutionary shifts toward malignancy [45,51]. This transition diverges into two distinct histopathological endpoints dictated by specific molecular trajectories and microenvironmental pressures.

CCC—the histotype most strongly associated with endometriosis [2,4,42]—is characterized by the early loss of the chromatin remodelling protein ARID1A and the subsequent activation of the PI3K/AKT/mTOR pathway via PIK3CA mutations [45,52,53,54]. The reported combination of ARID1A loss and PIK3CA activation appears to confer specialized metabolic fitness, allowing CCC to survive the high oxidative stress and iron-replete environment of the endometrioma, although the extent of these adaptations may vary between lesions [55].

In contrast, EnOC follows a trajectory of hormonal exploitation, frequently involving PTEN inactivation and KRAS or CTNNB1 mutations [45,53,56,57]. Unlike CCC, EnOC maintains robust estrogen receptor expression, utilizing the local hyperestrogenic milieu to drive unregulated mitogenic signalling and glandular proliferation. The presence of PTEN loss and KRAS mutations may differ across cohorts, potentially influencing clonal expansion and the establishment of hormonally responsive neoplastic clones [58,59]. Supporting a causal rather than merely associative relationship, a two-sample Mendelian randomization analysis utilizing genome-wide association study summary statistics demonstrated a significant genetically driven effect of endometriosis on ovarian cancer risk (OR = 1.19, 95% CI 1.11–1.29), with particularly strong associations for CCC (OR = 2.04, 95% CI 1.66–2.51) and EnOC (OR = 1.45, 95% CI 1.27–1.65), while no significant causal link with other cancer types was identified [37]. These findings provide robust epidemiological support for the histotype-specific oncogenic vulnerability observed in women with endometriosis, although the molecular determinants of individual susceptibility within this population remain to be fully characterized.

### 6.4. Epigenetic Mechanisms in Endometriosis-Associated Carcinogenesis

Epigenetic dysregulation represents a key driver of malignant transformation in endometriosis-associated ovarian cancer. Among these, loss of ARID1A—a core component of the SWI/SNF (SWItch/Sucrose Non-Fermentable) chromatin remodelling complex—is one of the most frequent and earliest events observed in both endometriosis and associated carcinomas, supporting a clonal relationship between precursor lesions and malignancy [60,61].

Primary studies have demonstrated that ARID1A deficiency leads to widespread epigenomic reprogramming, including altered chromatin accessibility and dysregulation of histone modifications-particularly H3K27 acetylation at enhancer regions [62]. Notably, ARID1A loss promotes hyperacetylation and activation of super-enhancers, resulting in increased transcriptional activity and enhancer RNA expression [63]. These effects are mediated, at least in part, by the histone acetyltransferase P300, whose activity is required to sustain the invasive phenotype of ARID1A-deficient endometriotic cells [62,64]. Targeting these epigenetic pathways may open new avenues for therapy, particularly in cases resistant to conventional treatments.

Downstream transcriptional programs are correspondingly affected, including activation of AP-1 signaling and upregulation of its JUNB subunit, which in turn regulates genes involved in EMT, extracellular matrix remodeling, and cell adhesion [65,66]. Functional studies have shown that JUNB is required for the invasive behaviour induced by ARID1A loss, directly linking epigenetic alterations to phenotypic changes characteristic of malignant progression [65].

Malignant transformation of endometriotic foci is further modulated by post-transcriptional mechanisms, among which dysregulation of the miR-200 family plays a central role [67]. These microRNAs, which normally contribute to the maintenance of epithelial characteristics and suppress EMT, become progressively downregulated or misexpressed in endometriotic tissues undergoing neoplastic transformation [68]. This shift facilitates the acquisition of invasive properties, promoting motility, extracellular matrix degradation, and early steps of metastasis [69]. Importantly, alterations in miR-200 expression appear to occur even in endometriotic lesions prior to overt carcinoma, suggesting that they represent early molecular indicators of malignant potential and a possible target for therapeutic intervention or biomarker development [67].

Collectively, these data support a model in which early genetic alterations, such as ARID1A mutation, drive epigenetic reprogramming of the chromatin landscape, leading to aberrant transcriptional activation and acquisition of invasive properties, thereby promoting the progression from benign endometriosis to carcinoma. By linking these molecular events to their clinical consequences, this review bridges mechanistic understanding with practical implications for patient care, surveillance, and treatment planning.

### 6.5. Clinicopathological Characteristics and Prognostic Implications

Despite this capacity for invasion, EAOC generally exhibit a more favourable prognosis compared to sporadic high-grade serous carcinomas (HGSC) [70,71]. This improved clinical outlook is largely attributable to earlier stage at diagnosis (Stage I/II), as symptoms related to pre-existing endometriomas—such as pelvic pain or adnexal masses—frequently prompt earlier surgical evaluation [71]. Biologically, EAOC typically follows the more indolent course characteristic of Type I tumors. The CCC histotype is notable for relative chemoresistance and an enhanced antioxidant response [55], whereas EnOC generally retains higher sensitivity to standard platinum-based regimens [71]. The distinct molecular profiles of EAOC not only define its unique etiology but also serve as potential biomarkers for targeted therapeutic strategies [39,45,67].

For instance, ARID1A loss may render tumors more susceptible to EZH2 inhibitors, offering a targeted approach that exploits the specific vulnerabilities arising from chromatin-remodeling deficiency. Similarly, activating mutations in *PIK3CA* nominate the PI3K/AKT/mTOR signaling pathway as a rational therapeutic target. *PTEN* loss underscores the continued relevance of hormone-modulating strategies, as these tumors may remain highly responsive to estrogen-driven signaling. Finally, *KRAS* mutations—more commonly observed in EnOC—may influence both chemotherapy responsiveness and long-term prognosis, suggesting that mutation-guided treatment selection could optimize patient outcomes [34,59,72,73].

Together, these molecular insights provide a nuanced framework for guiding patient-specific interventions. Integrating genomic profiling into clinical decision-making enables clinicians to tailor therapeutic approaches, anticipate resistance mechanisms, and personalize follow-up protocols according to the unique molecular landscape of each tumor—ultimately bridging the gap between mechanistic understanding and precision patient care.

## 7. Clinical Relevance: Surveillance and Follow-Up

The established link between endometriosis and EAOC carries significant implications for long-term clinical management, particularly given that these malignancies often arise at an earlier stage and in younger cohorts than sporadic ovarian cancers. Population-level screening for EAOC in women with endometriosis is not currently recommended; instead, clinical guidelines advocate for risk-adapted surveillance focused on patients with complex or persistent ovarian endometriomas [34,74]. This surveillance strategy requires multimodal integration of periodic imaging and biochemical monitoring. Longitudinal ultrasound and MRI are employed to detect subtle morphological or dimensional changes in ovarian lesions [75,76] while serial measurements of CA-125—despite its limited specificity—may indicate malignant transformation when a rapid, progressive rise is observed. Despite the availability of these non-invasive modalities, laparoscopy remains the gold standard for definitive diagnosis; excision and histological assessment of symptomatic or large endometriomas are essential to differentiate benign disease from borderline or invasive lesions [1]. To provide a practical overview of risk-adapted surveillance and management strategies for patients with endometriosis at potential risk of EAOC, a concise summary is presented in Table 4, integrating lesion characteristics, biomarker trends, and recommended clinical actions.

## 8. Molecularly Guided Clinical Work-Up: A New Frontier in Risk Stratification

The clinical management of EAOC is currently hampered by a “diagnostic gap,” wherein traditional imaging and biomarkers such as CA-125 frequently fail to detect early malignant transformation. To address this limitation, we propose an integrated molecular work-up that utilizes genomic findings to stratify risk and personalize surveillance (Table 4). Specifically, the identification of somatic driver mutations (e.g., ARID1A loss, PIK3CA or PTEN mutations) in endometriotic tissue or via liquid biopsy of cystic fluid provides a window of opportunity for early intervention. Patients harboring these “molecular red flags” should be transitioned from routine ultrasound follow-up to high-intensity surveillance protocols, including contrast-enhanced MRI and semi-annual monitoring. Furthermore, in cases of suspected malignant transformation, these molecular signatures could inform surgical strategy—favoring more radical, risk-reducing approaches over conservative cystectomy when the mutational burden suggests high oncogenic potential. This paradigm shift moves the field from a reactive “watch-and-wait” model toward a proactive, precision-medicine approach tailored to the individual genomic risk profile of each patient.

## 9. Research Agenda

On the basis of the available literature, several critical knowledge gaps remain, and further research is needed to advance both the mechanistic understanding and clinical management of endometriosis-associated ovarian cancer. The following priorities have been identified:

- **Validation of Early-Detection Biomarkers:** Priority must be given to validating non-invasive tools, such as miR-200 family profiling [67] and liquid biopsy. The goal is to identify molecular signatures of incipient malignancy before morphological changes become detectable on imaging.
- **Standardization of High-Risk Surveillance:** Prospective longitudinal studies are required to establish evidence-based protocols for high-risk cohorts (e.g., postmenopausal patients or those with large-diameter endometriomas). This includes defining “red flag” criteria to optimize the timing of prophylactic surgery.
- **Genomic Stratification in Surgical Care:** Research should investigate the utility of routine somatic mutational profiling (*ARID1A*, *PIK3CA*, *PTEN*) of endometriotic tissue. Identifying “pre-neoplastic” clones in benign lesions could guide personalized surgical counselling and earlier radical intervention [45,46].
- **Precision and Histotype-Specific Therapies:** Clinical trials are needed to evaluate targeted inhibitors of the PI3K/AKT/mTOR pathway specifically for CCC and EnOC histotypes [53]. Research should move beyond standard platinum-based chemotherapy to address the unique molecular vulnerabilities and chemoresistance of EAOC.

## 10. Limitations

This review, while comprehensive, is limited by the heterogeneity of primary studies and variability in reported mutation prevalence among endometriosis patients. Some mechanistic and therapeutic insights remain speculative, and rapidly evolving evidence may not yet be fully captured. Additionally, the integration of molecular, epigenetic, and clinical data relies on studies with differing methodologies and cohort characteristics. Despite these constraints, the review offers a coherent synthesis linking molecular mechanisms to clinical implications and surveillance strategies.

## 11. Conclusions

Endometriosis is increasingly recognized as a potential precursor of specific ovarian cancers, particularly EAOC. Chronic inflammation, estrogen exposure, and immune dysregulation promote a microenvironment that facilitates genomic instability and malignant transformation. Shared driver mutations such as ARID1A, PIK3CA, PTEN, and KRAS support a model of clonal progression from benign lesions to carcinoma. Although the absolute cancer risk remains relatively low, women with endometriosis have a significantly increased relative risk for these histotypes. Importantly, EAOC is often diagnosed at earlier stages and generally shows a better prognosis compared with other ovarian cancers.

Future perspectives should focus on validating non-invasive early-detection biomarkers, including circulating microRNAs and liquid biopsy approaches. In addition, integrating genomic profiling into clinical management and developing targeted therapies against pathways such as PI3K/AKT/mTOR may enable more personalized surveillance and treatment strategies.

## Figures and Tables

**Table 3:** Risk Stratification and Oncogenic “Red Flags” for Endometriosis-Associated Ovarian Cancer.

Category	Indicator of Increased Risk
Demographics	Age > 45–50 years; postmenopausal status [39,44]
Reproductive history	Nulliparity; late menopause; prolonged estrogen exposure [41,43]
Imaging red flags	Cyst diameter ≥ 9 cm; vascularized solid components; rapid growth [43]
Molecular drivers	*ARID1A* ^1^ loss-of-function; *PIK3CA* ^2^ activating mutations [45,46]
Histological risk	Presence of cytologic atypia in endometriotic lesions [32,39]

^1^ ARID1A: AT-rich interaction domain 1A; ^2^ PIK3CA: Phosphatidylinositol-4,5-bisphosphate 3-kinase catalytic subunit alpha.

**Table 4:** Risk-Adapted Surveillance and Management of Endometriosis-Associated Ovarian Lesions: Integrating Molecular and Clinical Indicators.

Feature/Risk Category	Surveillance & Diagnostic Action	Clinical Rationale & Molecular Insight
**Complex or persistent endometriomas**	TVUS every 6–12 months; MRI if morphology or vascularization changes.	Baseline monitoring for architectural stability [75,76].
**Rapid growth or morphological changes**	Contrast-enhanced MRI; consideration for early laparoscopic intervention.	Exclusion of occult malignant transformation or borderline lesions [43].
**Rising CA-125 (Serial increase)**	Serial measurements; laparoscopy indicated if levels show a progressive trend.	Marker of inflammatory flare or early neoplastic escape [1,25].
**Presence of Somatic Mutations** (*ARID1A*, *PIK3CA*, *PTEN*)	**Intensified surveillance:** High-resolution MRI every 6 months; NGS-based monitoring.	Molecular “red flags” indicating a high risk of clonal progression to EAOC [45,46].
**High Mutational Burden in Cystic Fluid**	**Liquid Biopsy:** Analysis of cfDNA in cystic fluid or blood to detect early genomic instability.	Minimally invasive detection of early-stage malignancy precursors [77,78].
**Symptomatic or large lesions (≥9 cm)**	Laparoscopic cystectomy with meticulous histological and molecular screening.	Definitive diagnosis; larger lesions provide greater surface area for transformation [24,43].
**High-risk patients** (Age > 45, Nulliparity, Family History)	Individualized, risk-adapted follow-up; consideration for risk-reducing surgery in post menopause.	Mitigation of cumulative estrogen-driven oncogenic risk [39,44].

TVUS: Transvaginal Ultrasound; MRI: Magnetic resonance imaging; NGS: Next-Generation Sequencing; EAOC: endometriosis-associated ovarian cancer; cfDNA: Circulating free DNA.

## Data Availability

The original contributions presented in the study are included in the article. Further inquiries can be directed to the corresponding author.

## Abbreviations

The following abbreviations are used in this manuscript:

EAOC	endometriosis-associated ovarian cancer
ARID1A	AT-rich interaction domain 1A
PIK3CA	Phosphatidylinositol-4,5-bisphosphate 3-kinase catalytic subunit alpha
PTEN	Phosphatase and tensin homolog
KRAS	Kirsten rat sarcoma viral oncogene homolog
RAS/MAPK	Rat sarcoma/Mitogen-Activated Protein Kinase pathway
PI3K-Akt-mTOR	Phosphatidylinositol 3-kinase Protein kinase B (Akt)—Mechanistic Target of Rapamycin pathway
rASRM	revised American Society for Reproductive Medicine classification
EFI	Endometriosis Fertility Index
SPE	superficial peritoneal endometriosis
DIE	deep infiltrating endometriosis
TVUS	Transvaginal Ultrasound
MRI	Magnetic resonance imaging
HOXA10	Homeobox A10
EOC	epithelial ovarian cancer
CCC	clear cell carcinoma
EnOC	endometrioid ovarian cancer
miRNA	microRNA
cfDNA	Circulating free DNA
